# Mast Cell-Derived Exosomes Promote Th2 Cell Differentiation via OX40L-OX40 Ligation

**DOI:** 10.1155/2016/3623898

**Published:** 2016-03-15

**Authors:** Fei Li, Yuping Wang, Lihui Lin, Juan Wang, Hui Xiao, Jia Li, Xia Peng, Huirong Dai, Li Li

**Affiliations:** Department of Laboratory Medicine, Shanghai First People's Hospital, Shanghai Jiao Tong University School, Shanghai 200080, China

## Abstract

Exosomes are nanovesicles released by different cell types, such as dendritic cells (DCs), mast cells (MCs), and tumor cells. Exosomes of different origin play a role in antigen presentation and modulation of immune response to infectious disease. In this study, we demonstrate that mast cells and CD4^+^ T cells colocated in peritoneal lymph nodes from BALB/c mouse. Further, bone marrow-derived mast cells (BMMCs) constitutively release exosomes, which express CD63 and OX40L. BMMC-exosomes partially promoted the proliferation of CD4^+^ T cells. BMMC-exosomes significantly enhanced the differentiation of naive CD4^+^ T cells to Th2 cells in a surface contact method, and this ability was partly inhibited by the addition of anti-OX40L Ab. In conclusion, BMMC-exosomes promoted the proliferation and differentiation of Th2 cells via ligation of OX40L and OX40 between exosomes and T cells. This method represents a novel mechanism, in addition to direct cell surface contacts, soluble mediators, and synapses, to regulate T cell actions by BMMC-exosomes.

## 1. Introduction

Exosomes are 30 to 100 nm extracellular membrane vesicles of endocytic origin, which are released into the extracellular environment upon fusion of multivesicular bodies with the plasma membrane. They were first reported* in vitro* in sheep reticulocytes by Johnstone et al. [1]. Subsequent reports showed that a range of cells including DCs, B cells, T cells, and tumor cells secreted exosomes* in vitro* and* in vivo*. Further, exosomes also occur naturally in body fluids such as blood, urine, saliva, and breast milk [2–6].

Although they share similar morphology, exosomes are unique in their protein composition. Because of their endosomal origin, all exosomes contain membrane transport and fusion proteins (GTPases, annexins, and flotillin), tetraspanins (CD9, CD63, CD81, and CD82), heat shock proteins (Hsp70 and Hsp90), proteins involved in multivesicular biogenesis (Alix and TSG), and lipid-related proteins and phospholipases [7, 8]. In addition to these membrane proteins, over 4400 different proteins have been identified in association with exosomes [9, 10]. Among these proteins, exosomes of different cellular origin or similar cells but in different states harbor different protein sets [11, 12]. For example, mast cell-derived exosomes carry c-kit and IgE receptors [13], and B cell-derived exosomes contain MHC II on the membrane [10]. Additionally, more MHC II, CD80, CD86, and other costimulatory molecules were detected in mature DCs-derived exosomes than in immature DCs [12]. A few studies suggested that the protein repertoire of exosomes was different from that of their parental cells [14]. Exosomes have been reported to contain significant amounts of RNAs, including miRNAs and mRNAs, but not DNA, and contain RNAs not detected in their parental cells [15].

Due to the existence of proteins and nucleic acid components, exosomes are supposed to play an important physiological and pathological role. In the last decade, much emphasis was placed on the effect of exosomes on immunological regulation and tumor development. Exosomes from mature DCs promoted the action of T cells, while those from immature DCs mediated immune tolerance [16]. Exosomes derived from bronchoalveolar lavage fluid suppressed allergic asthma in mouse models [17]. Tumor-derived exosomes promoted the proliferation of CD8^+^ T cells, which eliminated tumor cells [18]. These data indicate that exosomes contribute to the maintenance of health and are involved in the development of disease.

Mast cells are known as effector cells in the development of IgE-mediated allergic asthma. Degranulation after activation leads to secretion of bioactive substances, such as histamine, prostaglandin, and proteases to induce allergic response. Further, they also play a role in innate and adaptive immunity via secretion of cytokines such as TNF-*α*, IL-4, and IL-13. Mast cell-derived exosomes have been found to participate in immune regulatory response. To date, the effects of mast cell-derived exosomes on DCs, T cells, and B cells have been reported extensively. For example, mast cell-derived exosomes altered the phenotype of DCs* in vitro* [19]. In this study, we sought to determine the effects of exosomes from bone marrow-derived mast cells on naive T cells and the possible mechanisms.

## 2. Materials and Methods

### 2.1. Mice

BALB/c mice (5-wk-old) were purchased from Sion-British Sippr/BK Laboratory and housed in the Animal Experimental Center of Shanghai First People's Hospital (Shanghai, China) under specific pathogen-free conditions. The Chancellor's Animal Research Committee approved all the animal studies and confirmed that the experiments involving animals adhered to the guidelines set forth by the Shanghai Jiao Tong University School of Medicine (Shanghai, China).

### 2.2. Reagents and Antibodies

Fetal bovine serum (FBS), RPMI1640, and fluorescence dyes Dio and Dil were purchased from Life Technologies (California, USA). Recombinant mIL-3 and mIL-4 were purchased from PeproTech (Rocky Hill, NJ, USA). CD4^+^CD62L^+^ T cell Isolation Kit II was purchased from Miltenyi Biotec (Paris, France). FITC-labeled rat anti-mouse mAbs directed against CD117, PE-labeled rat anti-mouse mAbs directed against Fc*ε*RI, FITC-labeled rat anti-mouse mAbs directed against CD4, PE-labeled rat anti-mouse mAbs directed against IL-4, and PerCP/C*γ*5.5-labeled rat anti-mouse mAbs directed against IFN-*γ* were purchased from Biolegend (San Diego, CA). Goat anti-mouse OX40 mAb and rat anti-mouse OX40L mAb were obtained from R&D System (Minneapolis, MN, USA). Cell Counting Kit -8 (CCK-8, DojinDo, Japan) was used to assess the proliferation rate of cells. Antimast cell tryptase antibody was purchased from Abcam (America). Anti-rat IgG-HRP was purchased from Dako (Japan). ECL+ system was purchased from Amersham (Piscataway, NJ). All the information of primary antibodies is included in Table 1.

### 2.3. Preparation of Cells

BMMCs were prepared as previously described. After 4 wk of culture using RPMI 1640 supplemented with 10% heat-inactivated FBS and 10 ng/mL rIL-3, cells were harvested and consisted of 98% pure MCs as assessed by toluidine blue staining, CD117 and IgE receptor (Fc*ε*RI) expression. BMMCs were cultured for the last 72 h before harvest in the presence of RPMI1640 complemented with 10 ng/mL rIL-3 and FBS depleted of exosomes, allowing the use of supernatant containing only MC-derived exosomes.

Naive CD4^+^ T cells were negatively selected from the spleen cells by CD4^+^CD62L^+^ T cell Isolation Kit II. According to the manufacturer's instructions, the non-T-helper cells as well as regulatory T cells and *γ*/*δ*T cells were depleted by indirect magnetic labeling using a cocktail of lineage-specific biotin-conjugated antibodies against CD8a, CD45R, CD49b, CD11b, and Ter-119, as well as antibodies against CD25 and TCR*γ*/*δ* in combination with Anti-Biotin MicroBeads. Subsequently, CD4^+^CD62^+^ T cells were positively selected from the enriched CD4^+^ helper cell fraction with CD62L MicroBeads.

### 2.4. Exosomes Isolation

Exosomes were prepared from the supernatant of 4-wk-old BMMCs cultures [15]. During the last 72 h, BMMCs were cultured at 3 × 10^6^ cells/mL inIL-3-containing RPMI 1640. Supernatants were then subjected to two successive centrifugations at 300 g for 5 min and at 1,200 g for 20 min to eliminate cells and debris. Exosomes were purified by filtration of 0.22 *μ*g pore filters, followed by a centrifugation for 2 h at 120,000 g. Two fractions were obtained: a high-density (pellet) and a low-density (hypodense) fraction. The exosomes concentrated in the pellet were washed twice in a large volume of PBS centrifuged at 120,000 g for 2 h. The preparation of exosomes was stored under −80°C.

### 2.5. Coculture of Exosomes and Naive T Cells

Before coculture, 24-well plates were coated with anti-mouse CD3 and CD28 mAb both at 10 *μ*g/mL overnight at 4°C. 1 × 10^6^ naive T cells were incubated in 24-well plates with exosomes derived from 1 × 10^6^ or 1 × 10^7^ mast cells in 1 mL RPMI1640 supplemented with 10% exosomes-free FBS and 10 ng rlL-4 for 72 h. Alternatively, some groups were added into complete medium as blank control or 10 *μ*g/mL anti-mouse OX40L mAb according to the experiment. The assessment of proliferation of T cells was conducted by CCK-8 Kit according to manufacturer's protocol. In the last 4 hours, 100 ng PMA and 1 *μ*g A23187 were added into the medium, and, 2 hours later, 1 *μ*L GolgiStop was added to suppress the release of cytokines from plasma. In some experiments, naive T cells stained with Dio were cultured with exosomes stained with Dil.

### 2.6. Flow Cytometric Analysis

BMMCs were washed twice with PBS and stained with FITC-labeled CD117 and PE-labeled IgE receptor for 20 min under room temperature. Washed again, the samples were subjected to flow cytometry (Cytomics FC500, Beckman Coulter). For analysis of differentiation of T cells, cells were collected, washed with PBS twice, and stained with FITC-labeled anti-mouse CD4 mAb for 20 min under room temperature. Followed by fixation and permeabilization, intracellular cytokines IL-4 and IFN-*γ* were stained. Then FACS was performed to identify Th1 and Th2 cells.

### 2.7. Western Blotting

Exosomes were incubated for 30 min on ice in lysis buffer (PBS containing RIPA and protease inhibitors). In addition, cell lysates (1 million cells per 100 *μ*L of lysis buffer) from mast cells were also prepared. Insoluble material was removed by centrifugation (10,000 g for 10 min), and the protein concentration of supernatants was determined by protein assay. Protein concentrations of ultracentrifuged exosomes were again determined by BCA assay. Equal quantity of exosomes or cellular protein (5 *μ*g) was loaded per well of 10% acrylamide gels, and after electrophoresis proteins were transferred to PVDF membranes and blocked for 2 hours in PBS containing 3% BSA-TBST and 0.05% Tween-20. Primary antibodies were incubated for 2 hours and washed four times, followed by 1 hour of incubation with anti-rat IgG-HRP used at1 in 6,000 dilution in BSA-TBST. Detection of bands was performed with the ECL+ system.

### 2.8. Immunohistochemistry

Peritoneal lymph nodes were collected from healthy mouse and five-micrometer paraffin-embedded sections were prepared. Slides were stained with CD4^+^, tryptase, and DAPI recorded by fluorescence microscope (Zeiss). In addition, slides were, respectively, stained with CD4 and tryptase to assess T cells and mast cells.

### 2.9. Electron Microscopy

Exosomes were examined using negative staining as described in [20]. Briefly, the purified exosomes pellet was fixed in 2% paraformaldehyde at 4°C, and 5 *μ*L deposited on Formvar coated EM grids. After 20 min of absorption, grids were rinsed in PBS and fixed with 1% glutaraldehyde for 5 min. After washing in distilled water, grids were contrasted with uranyl oxalate (75 mM oxalic acid in 2% (w/v) uranyl acetate and final pH adjusted to 7.0 with NH_4_OH) and embedded in 4% (w/v) uranyl acetate and 2% (w/v) methylcellulose for transmission electron microscopy (Hitachi).

### 2.10. Statistical Analysis

Results were expressed as mean ± SD. Comparisons between mean values were performed by ANOVA, followed by the Student-Newman-Keuls (SNK) test. A value of *p* < 0.05 was considered significant.

## 3. Results

### 3.1. Colocalization of Mast Cells and CD4^*+*^ T Cells in Peritoneal Lymph Node

**Figure 1:**
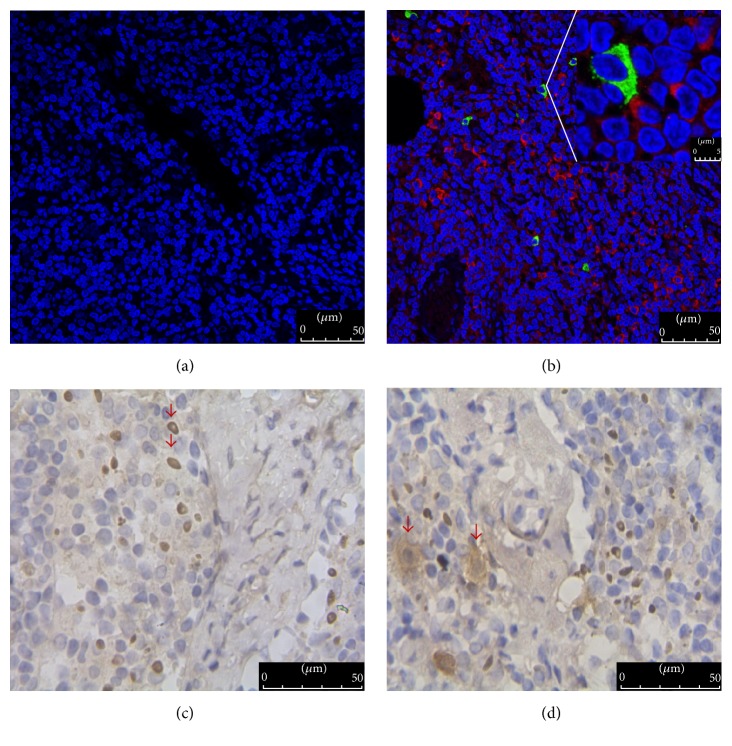
Location of mast cells and CD4^+^ T cells as well as their morphology in peritoneal lymph node. (a) As a negative control, the section of lymph node was incubated with PBS instead of primary antibody (200x); (b) mast cells (green, stained with antimast cell tryptase antibody) and CD4^+^ T cells (red, stained with anti-CD4 antibody) colocalized in the peritoneal lymph node, marked by the red arrows (200x); (c) as a control, the outline of CD4^+^ T cells is clear (400x); (d) mast cells are blurred and surrounded by tiny brown particles (400x). Scale bars are 50 *μ*m. In this experiment, CD4 and tryptase are used to mark CD4^+^ T cells and mast cells, respectively.

In previous studies, mast cells were associated with T cell activation in the immune response to resistant parasite infections as well as in allergic response [21, 22]. Further, these two cells were found to colocalize in intestinal tissues [23]. In the present study, we found that mast cells and CD4^+^ T cells coexisted in peritoneal lymph nodes of healthy mice and were closely linked (Figures 1(a) and 1(b)). When lymph node sections were stained with CD4 and tryptase, respectively, the shape of the CD4^+^ T cells was regular and clear, while mast cells were blurred, with brown particles observed outside the cells (Figures 1(c) and 1(d)). These data indicate that the mast cells potentially modulate the actions of CD4^+^ T cells.

### 3.2. Characteristics of Bone Marrow Mast Cells and Their Exosomes

**Figure 2:**
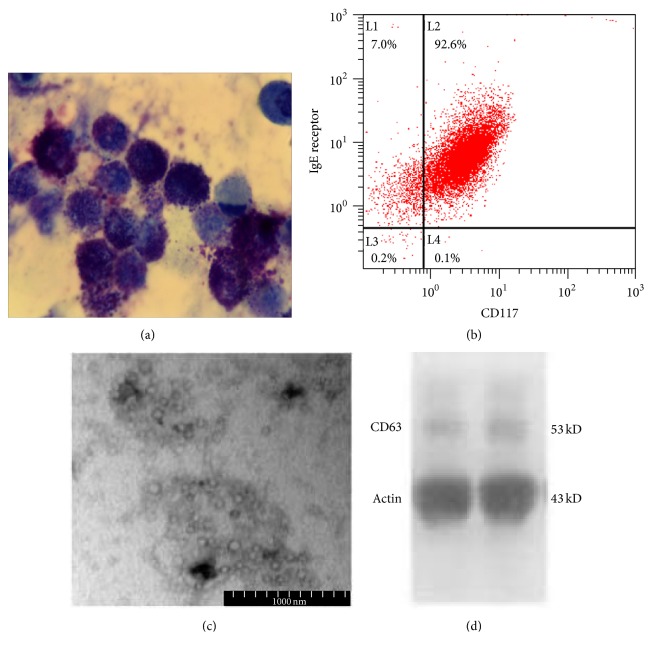
Characteristics of bone marrow mast cells and their exosomes. (a) Bone marrow mast cells showed abundant purple granules following toluidine blue staining (400x). (b) Mast cells were positive for CD117 and IgE receptor (Fc*ε*RI) by flow cytometry analysis. (c) BMMC-exosomes displayed a cup-shape morphology; scale bar = 100 nm. (d) Western blot indicated that BMMC-exosomes expressed CD63, a marker constitutively expressed by exosomes.

Mast cell-derived exosomes regulate immune response. In order to explore the effects of mast cell-exosomes on CD4^+^ T cells, mast cells from bone marrow were generated in the presence of IL-3 following a 4-week culture.  Figure 2(a) illustrates the morphology of BMMCs containing an abundance of purple granules after toluidine blue staining. Flow cytometry analysis was also performed to identify mast cells based on the expression of CD117 and IgE receptor, suggesting that over 98% of cells were mast cells (Figure 2(b)). The BMMC-exosomes were isolated by successive centrifugation. Electron microscopy showed that BMMC-exosomes were 60–100 nm in diameter and displayed a cup-shape morphology (Figure 2(c)).

The Western blot indicated that exosomes expressed CD63, a marker of membrane structure (Figure 2(d)). The data were consistent with the description of exosomes based on size and morphology, suggesting that BMMCs constitutively released exosomes.

### 3.3. BMMC-Exosomes Promote Th2 Differentiation

**Figure 3:**
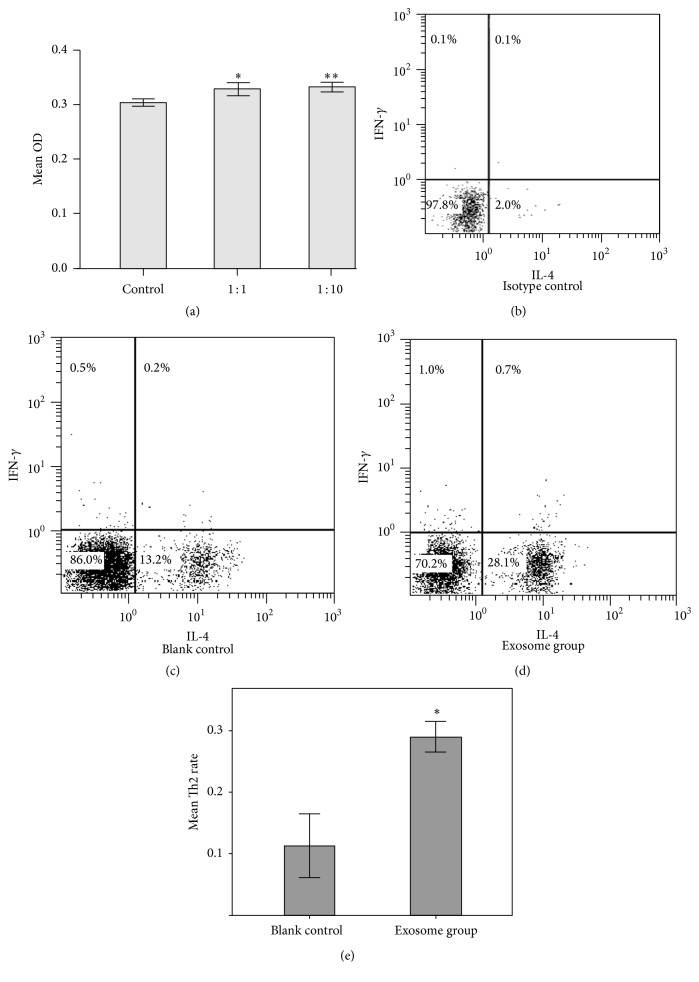
Effects of BMMC-exosomes on naive CD4^+^ T cells. (a) 10^6^ naive CD4^+^ T cells were coincubated with BMMC-exosomes generating 10^6^ or 10^7^ BMMCs under Th2 conditions. Compared with the blank control without exosomes, groups containing different levels of exosomes showed higher proliferation. T cell proliferation was quantified by incorporation of CCK-8. Results are expressed as mean O.D. at 450 nm and are representative of three experiments. *∗*, compared with control, *p* < 0.05. *∗∗*, compared with 1 : 1 group, *p* > 0.05. Student-Newman-Keuls (SNK) test was used. (b–d) After 72 h of culturing, the exosome group showed over 28% of Th2 cells, which was about twice the control group. (e) The rate of Th2 cells in the T cells was expressed as mean ± SD *∗*, compared with blank control, *p* < 0.05. The results represented four independent experiments.

To investigate the effects of BMMC-exosomes on the proliferation and differentiation of T cells, naive CD4^+^ T cells were coincubated with BMMC-exosomes in the presence of IL-4 (see Section 2.5) for 72 hours in 24-well plate precoated with anti-CD3 and anti-CD28 mAb. Compared with the blank control without exosomes in culture medium, T cells in the exosome group showed a higher proliferation rate at different exosome/T ratio (Figure 3(a)). In the following experiments, we used T cells and mast cells providing exosomes, in the ratio of 1 : 1. Surprisingly, we found that approximate 28% of cells were Th2 cells, which were IL-4 positive, while the blank control group constituted 13% of Th2 cells in the whole CD4^+^ cells (Figures 3(b)–3(e)). These results demonstrated that BMMC-exosomes promoted significant proliferation of T cells. Importantly, BMMC-exosomes enhanced the differentiation of naive CD4^+^ T cells to Th2 cells* in vitro*. These findings support the perspective that exosomes from mast cells modulated immune response.

### 3.4. BMMC-Exosomes Attached to CD4^+^ T Cells

**Figure 4:**
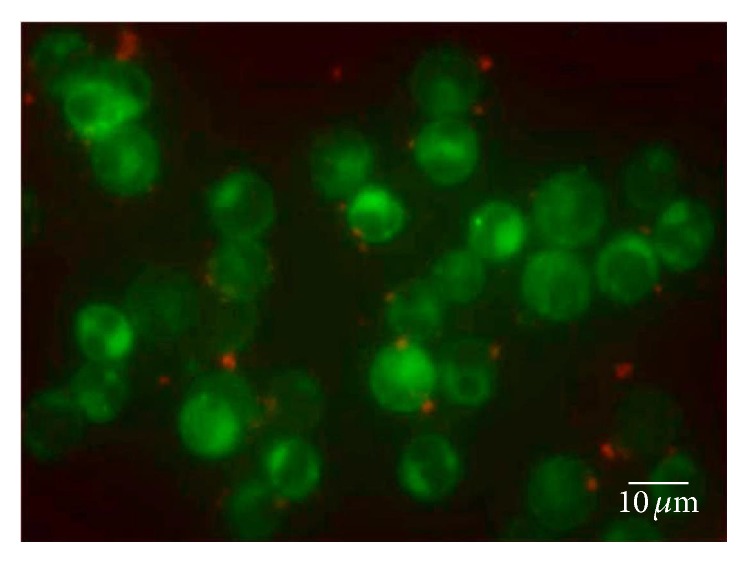
Mechanism of BMMC-exosome function in naive CD4^+^ T cells. Exosomes and T cells were stained with Dil (red-orange) and Dio (green), respectively. During the first 24 hours, exosomes were found to adhere to T cells and no endocytosis was observed. The picture was taken 12 hours after the coculture at a magnification ×400.

Previous studies reported that exosomes affected other cells in different ways. Morelli et al. reported that DCs internalized exosomes via endocytosis [24]. Another group found that DC-derived exosomes played a role in T cells by ligation of LFA-1 and ICAM-1 [25]. Further, exosomes may break down and release contents into the environment, to exert their functions. To clarify the association and localization of BMMC-exosomes within T cells, we labeled the T cells and exosomes with different fluorescent dyes and measured the dynamics over the first 24 hours. As shown in Figure 4, BMMC-exosomes adhered to the surface of T cells at 12 hours after coculturing. Even though the dish was swayed slightly, few exosomes separated from T cells. During the 24 hours, no exosomes were taken up by T cells. We, therefore, postulated that BMMC-exosomes may affect the action of T cells by direct surface contact or by release of their soluble contents, but not by endocytosis.

### 3.5. BMMC-Exosomes Function on CD4^+^ T Cells via Ligation of OX40L and OX40

**Figure 5:**
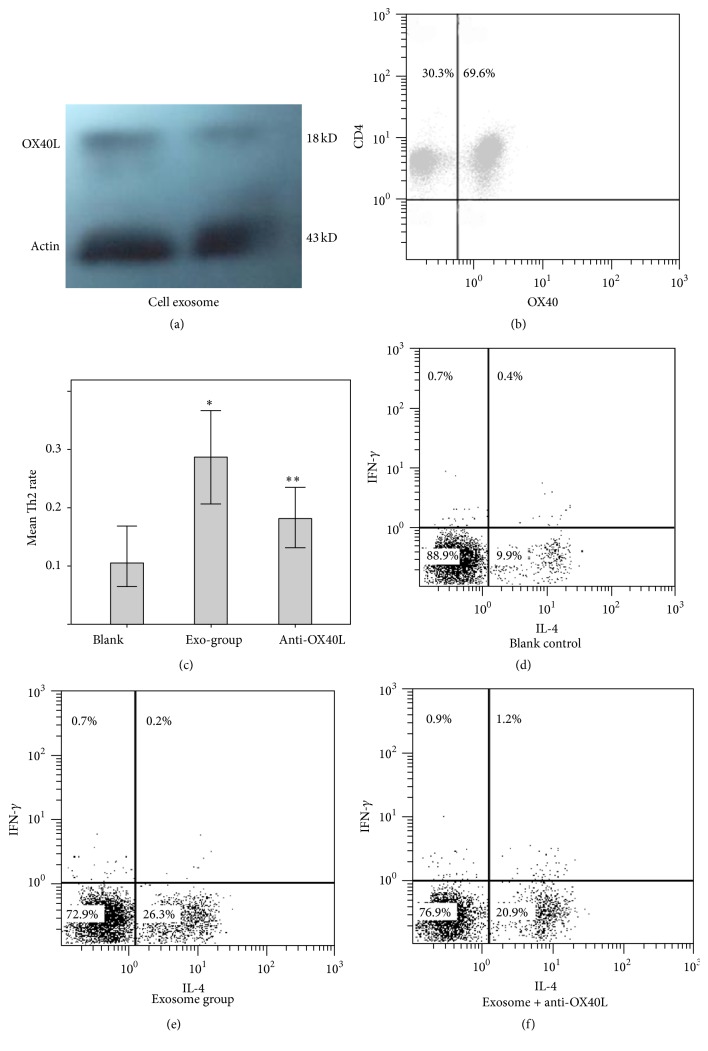
Ligation of OX40L and OX40 promotes the differentiation of Th2 cells. (a) Western blot revealed the expression of OX40L in BMMCs and their exosomes, but a lower expression on exosomes; (b) flow cytometry showed that naive CD4^+^ T cells expressed OX40; (c) Th2 expression in all the T cells was expressed as mean ± SD. *∗*, compared with blank control, *p* < 0.05. *∗∗*, compared with exosome group, *p* < 0.05. The results represented three independent experiments. (d–f) Addition of anti-OX40L Ab significantly but partially inhibited the differentiation of Th2 cells positive for IL-4.

We investigated the molecular mechanism by which BMMC-exosomes promoted the differentiation of naive CD4^+^ T cells to Th2 cells. Earlier studies demonstrated that IL-4, ICAM-1, CD40, and histamine facilitated differentiation of naive CD4^+^ T cells to Th2 cells. In addition, DCs enhanced the differentiation of Th2 cells via ligation of OX40L/OX40 between DCs and T cells [26]. MC activated T cells by OX40L [27]. Therefore, we wondered whether OX40L/OX40 played a role in the differentiation of T cells stimulated by BMMC-exosomes. The presence of OX40L was first determined in BMMC and BMMC-exosomes by Western blot, although it was lower in exosomes than in parental cells (Figure 5(a)). Further, OX40 was detected on the surface of T cells by FACS (Figure 5(b)). In the following experiments, anti-mouse OX40L mAb was added into the culture system to block OX40L on the exosomes. Compared with the exosome group, the group treated with exosomes and anti-OX40L mAb showed a lower number of Th2 cells. However, the inhibition of OX40L failed to completely inhibit the differentiation of Th2 cells to the level in the control group (Figures 5(c)–5(f)). These results demonstrated that OX40L on BMMC-exosomes ligated with OX40 on the surface of T cells to stimulate naive CD4^+^ T cell differentiate to Th2 cells. Other factors also promoted the differentiation of Th2 cells.

## 4. Discussion

Mast cells are ubiquitously distributed among tissues, including bone marrow and lymphoid tissue. For several decades, human mast cells have been established as the key effector cells in allergic inflammation. They bind IgE on their surface by expressing the high-affinity Fc receptor for IgE and release histamine and other mediators after crosslinking of surface-bound IgE by allergen. Currently, mast cells are viewed similar to lymphocytes and other major immune cells involved in host defense and homeostasis. Previous studies in mice and rats and to some extent in humans have shown that mast cells play a central role in host defense against bacteria and parasites, via the release of cytokines and other mediators that recruit neutrophils, eosinophils, and Th2 cells to the site of infection [28]. Further, mast cells have been identified in Zebrafish, with a hematopoietic system, which is highly conserved with human beings [29]. To date, few studies have reported allergic responses in fish. Therefore, mast cells may play other important roles in homeostasis, in addition to allergic inflammation. In this study, we found mast cells and CD4^+^ T cells colocalized in the peritoneal lymph node and attached to each other in some positions. Earlier reports also indicated that mast cells were found close to T cells at the intestinal tissue of patients with inflammatory bowel disease [23]. In addition, Wang et al. reported that mast cells migrated to lymph nodes in infections [30]. These findings suggested that mast cells reacted with T cells.

Referring to the interaction between mast cells and T cells, most investigators focused on mast cell function on T cells. Dimitriadou et al. found that mast cells captured antigens and presented them to T cells in MHC II-dependent manner, to activate T cells [31]. In another study, mast cells were reported to promote the differentiation of T cells to Th2 cells by release of histamine and TNF [32]. Since the discovery of exosomes, the effects of mast cell-derived exosomes on T cells have been explored in different conditions. When coincubated with spleen cells, mast cell-exosomes induced the activation of lymphocytes and production of IFN-*γ*, IL-2, and IgG [33]. In order to explore the effects of BMMC-exosomes on naive CD4^+^ T cells, we isolated naive CD4^+^ T cells using MACS column system and coincubated them with BMMC-exosomes. Interestingly, we found that BMMC-exosomes promoted differentiation of naive CD4^+^ T cells to Th2 cells significantly, when incubated with IL-4, which was not consistent with the results of Skokos et al. study [33]. The discrepancy may be attributed to the culture conditions. In our system, purified naive CD4^+^ T cells were incubated under Th2 condition, while Skokos et al.'s study incubated mixed spleen cells under neutral conditions without IL-4. In fact, we coincubated naive CD4^+^ T cells with BMMC-exosomes in the neutral condition, but no Th1 or Th2 cells were detected (data not shown). This result was consistent with the control group without exosomes and IL-4. Therefore, we speculated that the effect of BMMC-exosomes on T cells to a large extent depended on culture conditions.

For several decades, cell-cell communications were thought to occur by receptor-mediated events, either by recognizing a component of an adjacent cell surface, a transmitter from a synaptic partner, or via molecules released by other cells at varying distances. However, the discovery of exosomes changed the traditional theory. Evidence indicated that exosomes of different cellular origin acted on cells differently. For example, DCs stimulated proliferation of other T cells by engulfing T cell-derived exosomes [34]. Another report showed that DC-derived exosomes communicated with T cells by binding of LFA-1 and ICAM-1. Mittelbrunn et al. found that B cells transferred exosomes to T cells by immune synapse, which is a fairly surprising discovery [35]. In this study, we found that T cells did not engulf BMMC-exosomes, while a few exosomes adhered to T cells during the first 24 hours. Therefore, we speculated that BMMC-exosomes affected the differentiation of naive CD4^+^ T cells by surface contact. However, the mechanism involving breakdown of BMMC-exosomes, release of their intraluminal components into the culture, and impact on T cells cannot be excluded. Based on previous studies, ligation of OX40L and OX40 on T cells facilitated naive CD4^+^ T cells to differentiate to Th2 cells [26]. In addition, mast cells and their exosomes expressed OX40L. Therefore, we selected anti-OX40L mAb to block OX40L on BMMC-exosomes to significantly decrease their ability to promote the differentiation of Th2 cells, at least partly. Such a result demonstrated that ligation of OX40L and OX40 was the molecular mechanism by which BMMC-exosomes enhanced the differentiation of Th2 cells. Possibly, other surface molecules or intraluminal components of BMMC-exosomes also participate in the regulatory process. Therefore, we speculated that BMMC-exosomes may act on distant T cells and other cells, circulating in the blood stream or lymph system [5]. In contrast to cells, the small-sized exosomes migrate from the secretary sites to the remote sites quickly and easily. Unlike soluble cytokines distributed in distant locations at a low level, exosomes disintegrate to release the contents locally at a relatively high level to sustain their function. Therefore, exosomes may communicate more efficiently than cells and soluble mediators.

In conclusion, we found that BMMC-exosomes promoted the proliferation of naive CD4^+^ T cells, although the ability was limited. However, the exosomes significantly facilitated the differentiation of naive CD4^+^ T cells to Th2 cells by ligation of OX40L and OX40 between BMMC-exosomes and CD4^+^ T cells and represents a novel mechanism of cell-to-cell communication. The study limitation relates to the experimental conditions* in vitro* and the result may not completely represent the conditions* in vivo*. Therefore,* in vivo* studies are needed to elucidate the effects of MC-exosomes on CD4^+^ T cells.

## Figures and Tables

**Table 1:** Antibody profile.

Antibody	Company	ID number	Clone	Applications
Tryptase	Abcam	ab2378	AA1	IHC, IF
CD4	Biolegend	100401	GK1.5	IHC, FC, IP
CD4	Biolegend	100406	GK1.5	FC
CD117	Biolegend	105806	2B8	FC
Fc*ε*RI	Biolegend	134301	MAR1	FC
IL-4	Biolegend	144804	1015F8	FC
IFN-*γ*	Biolegend	127302	MAR1-5A3	FC
OX40	R&D	AF1256-SP	P47741	WB
OX40L	R&D	MAB1236-SP	182601	WB
*β*-actin	Proteintech	HRP-60008	ag0297	ELISA, WB

WB: Western blot; IHC: immunohistochemistry; FC: flow cytometry; IF: immunofluorescence; ELISA: enzyme-linked immunosorbent assay; IP: immunoprecipitation.
